# The learning curve in endoscopic transsphenoidal skull-base surgery: a systematic review

**DOI:** 10.1186/s12893-024-02418-y

**Published:** 2024-05-05

**Authors:** Abdulraheem Alomari, Mazin Alsarraj, Sarah Alqarni

**Affiliations:** 1 Neurosurgery Department, East Jeddah Hospital, 2277 King Abdullah Rd, Al Sulaymaniyah, 22253 Jeddah, Saudi Arabia; 2 Otolaryngology and Head and Neck Surgery Department, King Abdullah Medical Complex, Prince Nayef Street, Northern Abhor, 23816 Jeddah, Saudi Arabia; 3 Neurosurgery Department, King Abdulaziz Medical City, 21423 Jeddah, Saudi Arabia

**Keywords:** Endoscopy, Learning curve, Endonasal, Endoscopic skull base, Transsphenoidal surgery

## Abstract

**Background:**

The endoscopic endonasal transsphenoidal approach (EETA) has revolutionized skull-base surgery; however, it is associated with a steep learning curve (LC), necessitating additional attention from surgeons to ensure patient safety and surgical efficacy. The current literature is constrained by the small sample sizes of studies and their observational nature. This systematic review aims to evaluate the literature and identify strengths and weaknesses related to the assessment of EETA-LC.

**Methods:**

A systematic review was conducted following the PRISMA guidelines. PubMed and Google Scholar were searched for clinical studies on EETA-LC using detailed search strategies, including pertinent keywords and Medical Subject Headings. The selection criteria included studies comparing the outcomes of skull-base surgeries involving pure EETA in the early and late stages of surgeons’ experience, studies that assessed the learning curve of at least one surgical parameter, and articles published in English.

**Results:**

The systematic review identified 34 studies encompassing 5,648 patients published between 2002 and 2022, focusing on the EETA learning curve. Most studies were retrospective cohort designs (88%). Various patient assortment methods were noted, including group-based and case-based analyses. Statistical analyses included descriptive and comparative methods, along with regression analyses and curve modeling techniques. Pituitary adenoma (PA) being the most studied pathology (82%). Among the evaluated variables, improvements in outcomes across variables like EC, OT, postoperative CSF leak, and GTR. Overcoming the initial EETA learning curve was associated with sustained outcome improvements, with a median estimated case requirement of 32, ranging from 9 to 120 cases. These findings underscore the complexity of EETA-LC assessment and the importance of sustained outcome improvement as a marker of proficiency.

**Conclusions:**

The review highlights the complexity of assessing the learning curve in EETA and underscores the need for standardized reporting and prospective studies to enhance the reliability of findings and guide clinical practice effectively.

## Background

With the advent of endoscopic techniques, skull-base surgery has significantly advanced. The modern history of neuro-endoscopy began in the early 1900s with an innovation by Lespinasse and Dandy, involving intraventricular endoscopy to coagulate the choroid plexus for treating communicating hydrocephalus [1]. In 1963, Guiot first reported an endoscopic approach via the transsphenoidal route as an adjunct to procedures performed under microscopy [2, 3]. In 1992, Jankowski et al. described a purely endoscopic approach for pituitary adenoma resection [1].

The advantages of endoscopy have encouraged skull-base surgeons to adopt this technique, which provides a panoramic view of critical anatomical landmarks and improved access to the corners and deep surgical areas while inducing only minor trauma to the nasal structures, thereby enhancing postoperative patient comfort [4]. Compared with procedures involving microscopy, the endoscopic approach results in a shorter operating time (OT), a reduced hospitalization period, a lower rate of complications, and a higher endocrinological cure rate [5, 6]. Despite these benefits, the endoscopic approach is hindered by a two-dimensional view, instrument interference, difficulties in achieving homeostasis, and a steep learning curve (LC) [4].

Since its inception, pioneers in the field have recognized the steep LC associated with the endoscopic technique [7]. The safety and efficacy of the endoscopic endonasal transsphenoidal approach (EETA), as an alternative to the gold-standard microscopic technique, have been established. However, the steep LC associated with the endoscopic approach may affect short-term outcomes post-procedure [5, 6]. Additionally, as the skull-base endoscopic technique constantly evolves and expands, a thorough understanding of the associated LC is critical.

The results of existing publications on the EETA-LC are challenging to interpret due to small sample sizes, observational study designs, and a lack of standardization in assessment methodologies. In this systematic review aims to elucidate the EETA-LC from the literature by addressing the following questions: How was EETA LC evaluated? Which set of variables was used to assess the LC? What is the influence of the LC on the examined variables?

## Methods

A systematic review was conducted according to the Preferred Reporting Items for Systematic Reviews and Meta-Analyses guidelines [8]. The review was registered on PROSPERO (CRD42023494731). We searched different databases for articles that assessed the learning curve of EETA without date restriction (PubMed, and Google Scholar). We used a particular equation for each database using a combination of the following keywords and Medical Subject Headings: (Endoscopy OR endoscopic skull base OR endoscopic endonasal transsphenoidal approach) AND (Skull Base Neoplasms OR Pituitary OR pituitary adenoma) AND (Learning Curve OR endoscopic learning curve OR surgical learning curve).

First, two authors (AA, MA) independently screened the titles and abstracts of articles in the databases for learning curve analysis of EETA, either for a single surgeon or a team, by directly comparing outcomes between early and late cases performed. The full texts of the relevant articles were reviewed. When there was a disagreement, the articles were thoroughly discussed before their inclusion in the review. The bibliographies of the selected studies were also screened for relevant citations, which turned up studies that were already selected from the database search.

Studies were included according to the following inclusion criteria: 1) Comparison of outcomes between initial and advanced experiences with the endonasal endoscopic transsphenoidal approach to treat skull-base pathology, defined as "early experience" and "late experience," respectively; 2) Assessment of at least one parameter based on early and late experiences; 3) Randomized controlled trials, prospective cohort studies, retrospective cohort studies, case–control studies, and case series studies were included; and 4) English-language publications.

The study’s exclusion criteria included the following: 1) Studies not performing learning curve analysis; 2) Studies comparing the outcomes of microscopic and endoscopic transsphenoidal approaches without providing separate data for the endoscopic approach; 3) Studies comparing the learning curve between two EETA techniques, using simulated models or questionnaire-based analysis; 4) Studies comparing the microscopic vs. endoscopic approach without separate data available specifically for the endoscopic arm. Additionally, case reports, reviews, animal studies, technical notes, comments, and correspondence were excluded.

### Data collection and analysis

The following data were extracted directly from the articles: 1) author names; 2) the year of publication; 3) Time interval of performed procedures; 4) study design; 5) the sample size; 6) techniques used for learning curve analysis (methods used to assort the patients for the analysis); (conducting statistical analysis vs. simple comparison of outcomes); 7) the sample size in each study arm when group splitting performed (early experience vs. late experience); 8) detailed information about surgeon experience at the time of LC assessment (including or omitting the first few EETA cases); 9) single vs. multiple pathologies; 10) team vs. single-surgeon experiences; 11) evaluated set of variables; 12) Variables that improved with experience; and 13) the number of cases required to overcome the initial LC or other methods to identify overcoming the learning curve.

### Study quality assessment and risk of bias

Two reviewers conducted a quality assessment and evaluated the risk of bias in the included articles. We utilized the Newcastle–Ottawa Scale (NOS) [9] and the GRADE system [10].

Heterogeneity Analysis: Due to substantial heterogeneity observed among the included studies, which encompassed variations in study design, included pathologies, and outcome measures, a formal meta-analysis was not feasible. Therefore, we opted for a qualitative synthesis instead of a formal meta-analysis. Heterogeneity analysis and sensitivity analyses were not explicitly conducted.

## Results

**Fig. 1:**
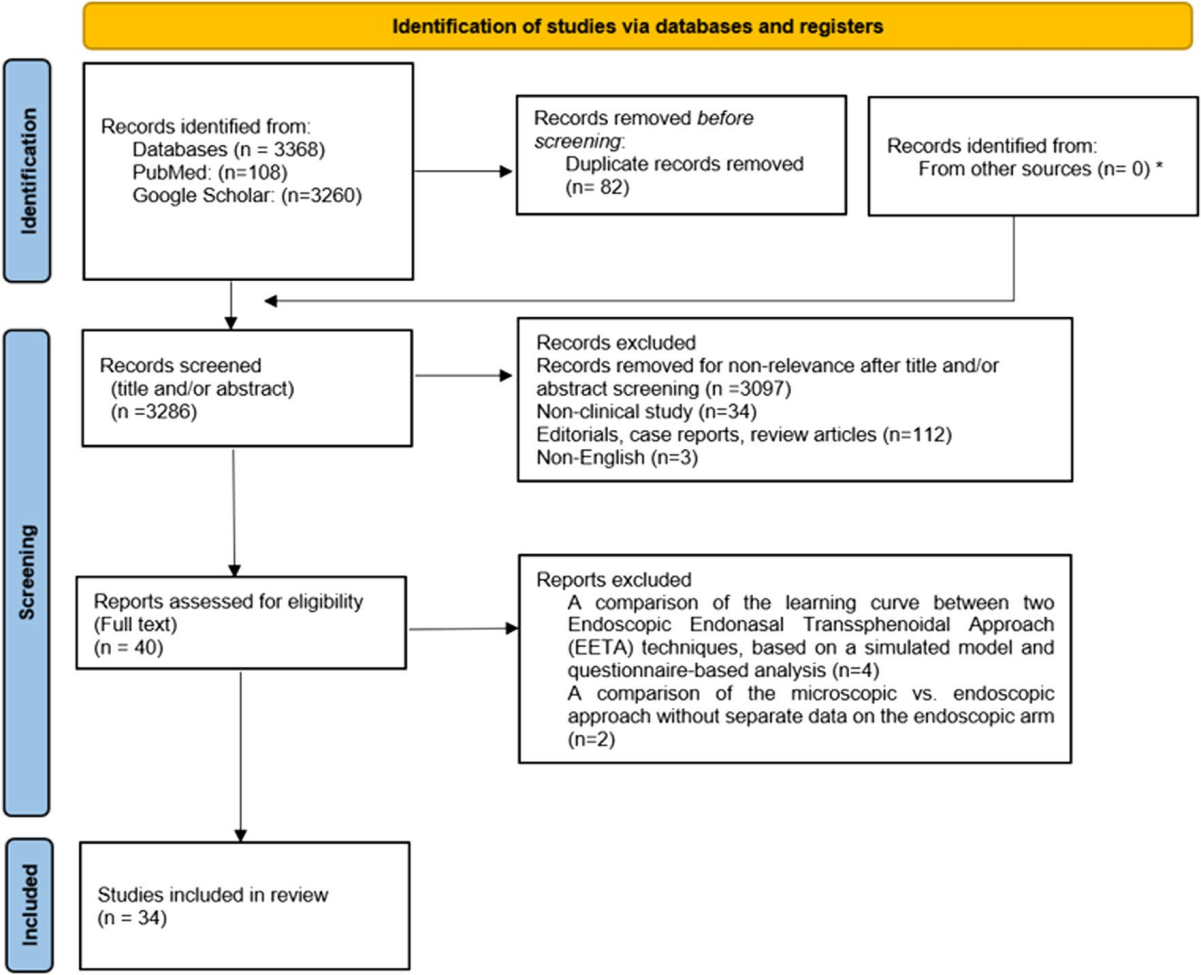
PRISMA flow diagram. PRISMA, Preferred Reporting Items for Systematic Reviews and Meta-Analyses * The bibliographies of the selected studies were also screened for relevant citations which turned up studies already included from databases search

Based on the inclusion and exclusion criteria, a total of 34 studies were identified (6 articles excluded after reviewing the full articles), including 5,648 patients [7, 11–43] (Fig. 1). The included studies were published between 2002 and 2022, and the evaluated procedures were performed between 1990 and 2018. The majority of the included articles comprised retrospective cohort studies (88%), with two being prospective studies, and two articles presenting data from both prospective and retrospective study designs. Assessing a surgical learning curve involves various methods and techniques documented within the included articles. We observed various methods for patient assortment in conducting learning curve analyses across the literature, with group-based learning curve analysis noticeable in a significant proportion of articles (68%). Within these studies, there was an unclear rationale behind patient grouping. Nonetheless, patients were categorized into either equal group, segmented based on arbitrary time periods, or separated based on improvements in outcomes observed retrospectively after data analysis. Eleven articles (32%) utilize case-based analysis, where individual surgical cases serve as distinct data points, and their outcomes are monitored over time.

Our systematic review encompasses a wide range of statistical tests employed in the included studies to analyze various data types and address multifaceted research inquiries. The primary statistical methodologies utilized encompass descriptive statistical analysis, which includes metrics such as mean, median, frequency, and standard deviation, along with comparative statistical analysis, which includes techniques such as Chi-square analysis, analysis of variance (ANOVA), and t-tests. Descriptive statistical analysis alone was evident in 10 articles (29%), whereas comparative statistical analysis was present in 24 articles (71%). Noteworthy examples include Leach et al. [16], who conducted analysis of variance (ANOVA) with post hoc Bonferroni tests for parametric data, Chi-Square Test, or Mann–Whitney tests for nonparametric data, and regression analysis to explore the relationship between surgical duration and relevant factors. Smeth et al. [17] undertook analyses using chi-square, Fisher exact, Student t-test, Mann–Whitney U test, and analysis of variance, aligning with their examination of categorical and continuous variables across distinct groups. Similarly, Sonnenburg et al. [12] applied a one-way ANOVA to discern variations between groups, highlighting the importance of understanding differences in means across categorical variables or treatment cohorts.

Regression analyses, scatterplots, McNemar tests, ROC curve analysis, and logistic regression models were integral across various studies, serving multiple purposes. Regression analyses, such as linear regression models, facilitated the exploration of intricate relationships among variables like age, tumor size, and surgical duration, identifying potential risk factors in surgical contexts [22]. Scatterplots visually depicted these relationships, offering intuitive insights into temporal variations, notably in the examination of surgery date versus duration [22]. McNemar tests were instrumental in evaluating changes in hormone levels, crucial for understanding postoperative outcomes and hormonal dynamics [37]. Additionally, ROC curve analysis provided a robust method for determining the level of surgical experience necessary to achieve gross total resection (GTR), offering actionable insights into surgical proficiency and patient outcomes [37]. Binary logistic regression models were utilized to identify prognostic factors contributing to the attainment of Gross Total Resection (GTR), hormonal recuperation, and visual restoration. For instance, variables such as surgical experience (≤ 100 vs. > 100 cases) were examined within this analytical framework [37].

In our examination of the included articles, we noted a lack of thorough description regarding the experience of surgeons or surgical teams with the endoscopic endonasal transsphenoidal approach (EETA), the extent of the approach undertaken, and the level of involvement of individual surgeons or surgical teams during procedures. Thirteen articles (38%) reported including the initial cases of EETA, which may indicate a lack of prior experience with the approach. Additionally, seven articles (21%) detailed the experience of a single surgeon, while the majority (79%) evaluated team experiences. There was a wide range of pathologies included in all the studies. Twenty articles (59%) focused on a single pathology, while fourteen studies (41%) examined multiple pathologies. Pituitary adenoma (PA) was the most frequently reported pathology (82%), followed by craniopharyngioma (CP) (44%). Three studies assessed the learning curve of cerebrospinal fluid (CSF) leak repair following treatment of multiple pathologies. Descriptions of the surgical approach, particularly distinguishing between simple and extended techniques, were notably lacking across all articles. However, seventeen articles (50%) did mention pathologies that often require an extended approach, such as meningioma, chordoma, and CP. A number of studies have investigated the variations in tumor type and size among the examined groups, particularly between early and late groups. Notably, findings from studies such as [7, 16, 17, 22, 23, 26, 38] indicated that no statistical differences were observed between these groups. The characteristics of the included studies [7, 11–43] are summarized in Table 1.

**Table 1:** Summary of eligible studies

Author	Study design	Patients’ age range (years)	Number of patients	Patients in each group	First few cases included	Pathology (single or multiple)	Team or single surgeon	Descriptive or comparative SA^a^
Younus et al. [7]	Retrospective	NA	1,000	500/500	No	PA,CP,others	Team	Comparative SA
Cappabianca et al. [11]	Retrospective	17–75	100	50/50	Yes	PA, CP, others	Team	Descriptive SA
Sonnenburg et al. [12]	Retrospective	NA	45	15/15/15	Yes	PA, RC, others	Team	Comparative SA
Kenan et al. [13]	Retrospective	11–67	78	40/38	Yes	PA	Team	Comparative SA
Yano et al. [14]	Retrospective	15–85	233	NA	NA	PA, CP, others	Team	Comparative SA
Gondim et al. [15]	Retrospective	13–79	228	NA	Yes	PA	Team	Descriptive SA
Leach et al. [16]	Retrospective	NA	125	53/72	Yes	PA, CP, others	Single surgeon	Comparative SA
Smith et al. [17]	Retrospective	NA	51	17/17/17	Yes	PA, CP, others	Team	Comparative SA
Wagenmakers et al. [18]	Retrospective	NA	36	NA	NA	PA	Team	Comparative SA
Kumar et al. [19]	Retrospective	18–83	136	68/68	Yes	PA, CP, others	Team	Descriptive SA
Snyderman et al. [20]	Retrospective	NA	700	NA	Yes	PA, CP, others	Team	Descriptive SA
Bokhari et al. [21]	Retrospective	26–85	79	27/26/26	Yes	PA	Single surgeon	Comparative SA
Chi et al. [22]	Retrospective	21–78	80	40/40	Yes	PA	Single surgeon	Comparative SA
de los Santos et al. [23]	Retrospective	32–84	40	20/20	Yes	PA,AC,others	Team	Descriptive SA
Hazer et al. [24]	Retrospective	17–75	217	NA	No	PA	Team	Comparative SA
Jakimovski at el. [25]	Prospective	NA	203	NA	NA^b^	PA	Team	Comparative SA
Koutourousiou et al. [26]	Retrospective	27–88	45	15/15/15	No	Meningioma	Team	Descriptive SA
Mascarenhas et al. [27]	Retrospective	5–86	122	63/63 (126 surgeries)	NA	PA,CP,others	Team	Descriptive SA
Ottenhausen et al. [28]	Retrospective	31–81	20	8/12	No	Meningioma	Team	Comparative SA
Ananth et al. [29]	Retrospective & prospective	19–62	32	NA	NA	PA,CP,others	Single surgeon	Descriptive SA
Jang et al. [30]	Retrospective	21–78	331	102/229	NA	PA	Team	Descriptive SA
Kshettry et al. [31]	Retrospective	14–74	43	20/23	NA	CP	Team	Comparative SA
Qureshi et al. [32]	Retrospective	28–80	78	9/69	NA	PA	Team	Comparative SA
Shou et al. [33]	Retrospective	14–74	178	89/89	NA	PA	Team	Comparative SA
Ding et al. [34]	Retrospective	21–67	33	17/16	NA	CP	Team	Comparative SA
Shikary et al. [35]	Retrospective	NA	202	NA	Yes	PA	Team	Comparative SA
Eseonu et al. [36]	Retrospective & prospective	NA	275	118/157	NA	PA	Single surgeon	Comparative SA
Kim et al. [37]	Retrospective	16–86	331	100/231	NA	PA	Single surgeon	Comparative SA
Lofrese et al. [38]	Retrospective	42–65	95	47/48	NA	PA	Team	Comparative SA
Robins et al. [39]	Retrospective	NA	142	NA	NA	PA	Team	Comparative SA
Algattas et al. [40]	Retrospective	3–82	62	NA	No	CP	Team	Descriptive SA
Soliman et al. [41]	Retrospective	27–82	56	NA	NA	PA,CP,others	Team	Comparative SA
Nix et al. [42]	Prospective	2–81	127	45/82	NA	PA,CP,others^b^	Team	Comparative SA
Park et al. [43]	Retrospective	NA	125	30/95	Yes	Meningioma, CP, others^b^	Single surgeon	Comparative SA

*NA* Not available, *PA* Pituitary adenoma, *CP* Craniopharyngioma, *RC* Rathke’s cyst, *SA* Statistical analysis performed

^a^The utilization of either comparative statistical analysis or solely descriptive statistical analysis

^b^Learning curve of cerebrospinal fluid leaks repair (spontaneous or following resection of multiple pathologies)

**Fig. 2:**
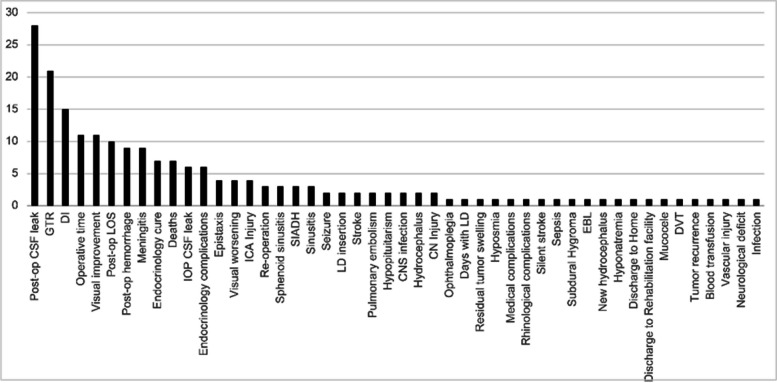
Frequency at which certain variables were evaluated in the literature to assess the EETA learning curve. EETA, endoscopic endonasal transsphenoidal approach; post-op, postoperative; CSF, cerebrospinal fluid; GTR, gross total resection; DI, diabetes insipidus; LOS, length of stay; IOP, intraoperative; ICA, internal carotid artery; SIADH, syndrome of inappropriate antidiuretic hormone secretion; LD, lumbar drain; CNS, central nervous system; CN, cranial nerve; EBL, estimated blood loss; DVT, deep vein thrombosis

The EETA-LC was evaluated based on a diverse set of variables. The most frequently analyzed variables were postoperative cerebrospinal fluid (CSF) leak in 28 articles (82%) [7, 12, 13, 15–17, 19–23, 25, 27–29, 31–43], gross total resection (GTR) in 21 articles (62%) [7, 13, 14, 16, 19, 21, 22, 26–34, 36–40], post operative diabetes insipidus (DI) in 15 articles (44%) [12, 13, 16, 17, 19, 21, 22, 29–32, 34, 36, 37, 41], operative time (OT) in 12 articles (35%) [7, 13, 14, 16, 17, 22, 29, 32, 34–36, 38] and visual improvement in 12 articles (35%) [13, 14, 16, 21, 22, 28, 31, 32, 34, 36, 37, 41]. (Fig. 2).

**Fig. 3:**
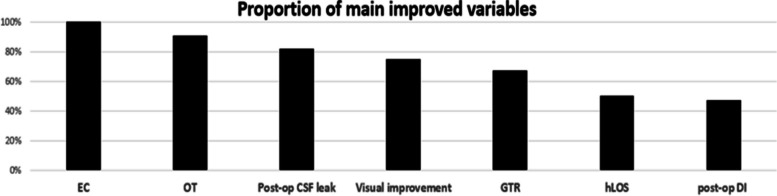
Proportion of main improved variables with experiences. EC, Endocrinological cure; OT, Operative time; post-op: postoperative; CSF, cerebrospinal fluid; GTR, gross total resection; hLOS, hospital length of stay; DI, diabetes insipidus

In all the studies included, improvements were observed between early and late-experience stages [7, 11–43]. Among the evaluated variables, the following improvements were noted: the endocrinological cure rate (EC) showed improvement in all 7 articles out of 7 evaluated [13, 16, 18, 21, 24, 30, 33], operative time (OT) improved in 11 out of 12 articles (91%) [13, 14, 16, 17, 22, 29, 32, 34–36, 38], postoperative cerebrospinal fluid leak (CSF) improved in 23 out of 28 articles (82%) [12, 15, 17, 19, 20, 22, 23, 25, 27–29, 31–35, 37–43], visual improvement was observed in 9 out of 12 articles (75%) [13, 14, 16, 22, 28, 31, 34, 37, 41], gross total resection (GTR) improved in 14 out of 21 articles (67%) [7, 13, 14, 19, 21, 22, 26–30, 38–40], hospital length of stay (LOS) decreased in five out of 10 studies (50%) [11, 12, 16, 17, 22], and postoperative diabetes insipidus (DI) decreased in 7 out of 15 articles (47%) [3, 14, 16, 17, 21, 22, 33] (Fig. 3).

Moreover, 12 articles (35%) reported both significant and non-significant improvements in outcomes [7, 13, 14, 16, 17, 21, 22, 31, 32, 34, 38, 41]. In 10 studies (29%), solely a trend of improvement was observed [11, 15, 19, 20, 23, 26, 27, 29, 30, 40], while 8 articles (23%) reported solely significant improvements [18, 24, 25, 35–37, 42, 43]. However, in four studies, despite observing a tendency towards better outcomes, no statistical disparities were identified among all assessed variables [12, 28, 33, 39]. None of the included studies reported a deterioration in any of the assessed outcomes over time, except for one study where a significant decline in GTR was observed in the late group [33]. This decline was attributed to the inclusion of more invasive and complex tumors in the late group. Nevertheless, Younus et al. documented ongoing improvement in GTR even after surpassing the initial learning curve [7].

In this systematic review, the primary technique employed to determine the transition point indicating the overcoming of the initial learning curve involved observing sustained and consistent improvement in outcomes over time. In almost half of the included articles, overcoming the initial learning curve (observing improvement of outcomes) was linked to the number of cases performed. Out of the 34 analyzed studies, 16 (47%) estimated the number of cases needed to overcome the initial learning curve of EETA. Reported cases ranged widely from 9 to 120, with a mode of 50. Considering both the median and the Interquartile Range (IQR) provides a comprehensive understanding of the reported case distribution and central tendency for overcoming the initial EETA learning curve. The median number of cases needed is 32, with an IQR of 20. These numbers are estimates and require careful interpretation [16, 17, 20–25, 29, 31–33, 35–38, 42].

Regarding the quality of included studies, the NOS quality assessment scale was used. 21 studies graded as fair quality while the remaining 13 articles rated as poor quality [9]. The risk of bias was evaluated according to the GRADE system. All included studies are observational cohort study and graded either as low or very low grade [10]. This reflects the great heterogeneity and high risk of bias due to the study design of the current EETA-LC literature.

## Discussion

Endoscopic techniques have drastically improved skull-base surgery. Unlike procedures involving a microscope, many neurosurgeons have acquired experience in endoscopic techniques later in their careers, and the level of exposure to these techniques during training years has varied among surgeons. The LC is a critical factor in the acquisition of new surgical skills. Understanding the link between the EETA-LC and surgical outcomes will enable surgeons to better understand what to expect and what measures to apply as those surgical skills develop. Many studies in other surgical domains have reported on the LC during the acquisition of new surgical techniques [44–47]. Most minimally invasive surgeries are associated with a challenging LC, and EETA is no exception [7, 46].

The concept of the LC was first established in the field of aircraft manufacturing and refers to an improvement in performance over time [48]. Smith et al. [17] have defined it as the number of procedures that must be performed for the outcomes to approach a long-term mean rate. Typically, an LC is characterized by an S-shaped curve with three stages: an early phase, during which new skill sets are acquired; a middle phase, in which the speed of learning rapidly increases; and an expert phase in which the performance reaches a plateau [49]. However, other curves have been proposed that involve a dip in the LC following the initial acceleration of the learning rate; this occurs especially with handling more challenging cases. Another potential decline may emerge after a long period of experience. Despite having reached a plateau in the learning curve after an extended period, declines in manual dexterity, eyesight, memory, and cognition may overshadow the benefits of accumulated experience, leading to diminished performance levels [50].

The absence of consensus on the best applicable methods to describe and assess the learning curve may explain the diversity of analysis methods observed in this systematic review. In their large systematic review regarding learning curve assessment in healthcare technologies, Ramsay et al. [51] reported that group splitting was the most frequent method. They defined group splitting as dividing the data by experience levels and conducting testing on discrete groups, often halves or thirds. The statistical methods applied included t-tests, chi-squared tests, Mann–Whitney U tests, and simple ANOVA.

In our review, we reached a similar conclusion. We observed that a substantial portion of articles (68%) utilized group-based learning curve analysis [7, 11–13, 16, 17, 19, 21–23, 26–28, 30–34, 36–38, 42, 43]. Additionally, we similarly noted that papers frequently lacked explanations for the selection of cut points, raising concerns about potential bias resulting from data-dependent splitting. It is important to acknowledge that this method of group categorization has inherent drawbacks, including challenges related to small sample sizes, the use of arbitrary cutoff points, and the inability to eliminate all potential confounding variables [52].

Descriptive analysis was found in 10 articles (29%) within this review [11, 15, 19, 20, 23, 26, 27, 29, 30, 40]. While providing an initial grasp of data distribution and characteristics, descriptive analysis may fall short in capturing the intricate dynamics of the learning curve over time or the factors affecting its impact [51]. Alternatively, conducting rigorous statistical analyses afterward offers better insight and interpretation of the results. This approach aims to mitigate the influence of confounding factors on outcome assessments over time [51, 52].

In our review, 24 articles (71%) conducted a wide variety of statistical analyses [7, 12–14, 16–18, 21, 22, 24, 25, 28, 31–39, 41–43], including but not limited to the following tests: Chi-square Test, Fischer exact test, Student's t-test, Analysis of Variance (ANOVA), Mann–Whitney U Test, McNemar tests, Multivariate linear regression model, Cumulative Sum (CUSUM), and ROC Curve Analysis [13, 16, 22, 32, 37–39]. Four studies indicated that there was no statistically significant difference observed among the variables under evaluation. The lack of significance was attributed to several factors including small sample sizes, meticulous case selection, involvement of an otolaryngology team throughout the procedure, an increase in the number of invasive tumors in the late-experience study group, previous surgical experience, intensive training, level of supervision, and gradual inclusion of residents [12, 28, 33, 39]. These efforts should be regarded as beneficial strategies aimed at reducing the steepness of the EETA learning curve.

To obtain more accurate results, it is crucial to eliminate confounding factors, such as the level of supervision, prior experience, the heterogeneity of cases being treated, and their complexity when evaluating the LC. Thus, it is essential to incorporate multivariate logistic regression analysis to mitigate the impact of these potential confounding factors [51]. Chi et al. [22] divided their patients into equal groups of 40 cases each. They then compared potential confounding variables to minimize their influence on learning curve assessment. This comparison includes demographic and clinical factors between the two groups, such as sex distribution, mean age, tumor size (microadenomas vs. macroadenomas), visual field defects, and tumor types (non-functioning, functioning adenomas, etc.). By conducting these comparisons, the researchers sought to identify discrepancies in demographic and clinical features between the groups.

The description of a surgeon's extensive prior experience is crucial for accurately quantifying the assessment of the learning curve, a point reported to be neglected during the assessment in various types of learning assessments related to healthcare procedures [49]. In our review, we observed the same conclusion in all included studies. However, the inclusion of the initial first few cases was mentioned in 13 (38%) articles, which might be used as a surrogate for no prior experience with EETA. Furthermore, five articles did not include the initial few cases. Among these, four studies examined the learning curve of more complex cases such as meningioma, craniopharyngioma, and growth hormone pituitary adenoma, employing an extended approach. Conversely, Younus et al. [7] deliberately excluded these cases to assess various stages of the learning curve.

Assessing multiple pathologies with varying complexities could significantly impact learning curve assessments. In our review, 59% of articles focused on a single pathology, while 41% explored multiple pathologies. Pituitary adenoma (PA) was the most evaluated (82%), followed by craniopharyngioma (CP) (44%). Controlling confounding variables like tumor type and size may yield more reliable results. Some studies used statistical analyses to compare early and late cases, while others relied on descriptive analyses. Shou et al. noted a drop in GTR over time due to late involvement of complex cases [33]. Conversely, studies analyzing tumor size and type found GTR improvement with experience [7, 23]. Thorough multivariable analysis of confounding factors is crucial for representative LC analysis.

The LC is often assessed based on two main categories of variables: those related to the surgical procedure (OT, estimated blood loss, and extent of resection) and those related to patient outcomes (duration of hospitalization, the incidence of complications, and the mortality rate) [50]. In this systematic review, OT was one of the most frequent parameters that significantly reduced as one gained experience. Although OT is commonly utilized as an outcome measure, it is only a surrogate means of evaluating the LC and may not always accurately represent patient outcomes [52]. Another point to consider is the lack of standardized variables for assessing the LC, and the included studies evaluated more than 45 distinct variables. Khan et al. highlighted the importance of using consistent variable definitions across studies to derive accurate conclusions from aggregated LC data [52].

A dynamic relationship exists between surgical outcomes and the LC, and each phase of the LC influences a distinct set of variables differently. One study, which included data from 1,000 EETA cases after purposely eliminating the first 200 cases, showed that variables such as GTR and the endocrinological cure rate continued to improve after the first 200 cases, whereas other parameters remained unchanged. Authors concluded that some variables will continue to improve after passing the initial LC phase [7]. Determining the precise number of cases needed to surpass the initial learning curve (LC) has proven challenging. Shikary et al. observed a notable decrease in postoperative CSF leaks after 100 surgeries, while a reduction in operative time was evident after 120 cases [35]. However, specifying a definitive number to overcome the learning curve of the Endoscopic Endonasal Transsphenoidal Approach (EETA) remains challenging due to individual variability, diverse pathologies, and evolving surgical techniques.

Assessing the learning curve of the Endoscopic Endonasal Transsphenoidal Approach (EETA-LC) faces notable challenges due to its intricate techniques and the wide array of pathologies it addresses. The diversity across specialties makes standardizing studies difficult. To understand the dynamic learning process in EETA-LC, influenced by individual surgeon skill, patient nuances, and procedural complexities, longitudinal studies and advanced analytical methods are essential. Moreover, the complexity of statistical analysis adds another layer of challenge, highlighting the necessity for interdisciplinary collaboration and innovative methodologies.

To address the current limitations in the literature regarding the EETA LC, we propose several key strategies for future studies. Firstly, we advocate for multicenter collaboration, coupled with standardized processes, to comprehensively assess the EETA LC. This collaborative approach will facilitate the aggregation of data from diverse surgical settings, enhancing the generalizability of findings and minimizing bias. Furthermore, rigorous documentation of the previous and current experience of involved surgeons is paramount. We suggest categorizing surgeons based on their levels of experience to accurately elucidate the impact of proficiency on surgical outcomes. Secondly, given the wide variety of complexities of skull base pathologies encountered, we recommend further categorization of cases based on their levels of complexity. This stratification will enable a more nuanced analysis of the learning curve across different levels of surgical challenge. Thirdly, standardization of outcome measures used to assess the learning curve is imperative, with specific definitions provided for each outcome. This ensures consistency and comparability across studies, facilitating meaningful interpretation of results. Finally, conducting prospective study designs with sufficient follow-up periods, along with rigorous multivariate statistical analyses among these categorized groups, is essential to mitigate the influence of confounding variables and strengthen the validity of findings. Implementing these strategies will help future studies to overcome the current limitations in the literature, leading to a deeper understanding of the EETA learning curve and ultimately improving patient outcomes.

## Conclusions

This systematic review identified 34 studies that reported a relationship between improvements in surgical outcomes and a surgeon’s level of experience with EETA. There is notable significant heterogeneity in the current literature on EETA-LC regarding the techniques used to assess the LC, variables assessed, types of pathology included, and insufficient reporting of the surgeon or team's current and previous experience with EETA. The main variables improved with experience were EC, postoperative CSF leak, OT, GTR visual improvement, and hospital LOS. Future studies with multicenter collaboration and standardized processes for assessing the EETA LC will enhance generalizability and minimize bias. Rigorous documentation of surgeons' experience levels, categorization of cases by complexity, and standardized outcome measures are essential. Additionally, rigorous statistical analyses will strengthen validity and mitigate confounding variables. Implementing these strategies will deepen our understanding of the EETA learning curve, ultimately leading to improved patient outcomes.

## Data Availability

The datasets used and/or analysed during the current study are available from the corresponding author on reasonable request.

## Abbreviations

EETA: Endoscopic endonasal transsphenoidal approach
LC: Learning curve
CSF: Cerebrospinal fluid
DI: Diabetes insipidus
GTR: Gross total resection
